# Chatham Army Medical Museum Reports

**Published:** 1839-01

**Authors:** 


Art. III.—*The first Fasciculus, second Fasciculus, and third Fasciculus of Anatomical Drawings, selected from the Collection of Morbid Anatomy in the Army Medical Museum at Chatham.—London,* 1824, 1834, 1838. Imperial folio.

The first of these fasciculi was published in 1824; the second and third at intervals of ten and four years respectively. The selection of the subjects is judicious, and the artistical execution, particularly of the two last, sufficient for all the purposes which uncoloured representations of disease can serve,—viz. to give a more clear conception of its anatomical characters than can be conveyed by any description of them, however detailed. We, however, never can look at such representations of morbid structure without feeling strongly their deficiency from want of colour, for which no description can be an adequate substitute; and we feel this deficiency the more in the plates before us, that no allusion is made to this most important physical character of the diseases described; and that no attempt has been made to increase the value of the plates by letters of reference to the most important parts of the disease represented in each figure. We notice this latter defect, as it may easily be remedied in the succeeding fasciculi; and we would also suggest that more care be taken in correcting the letter-press, as an error has been committed in the numbering of some of the figures in plate ii., second fasciculus; and a much more serious one in the description of fig. 3, in which it is stated that pulmonary emphysema "consists in large *cul-de-sac* dilatations of the extremities of the bronchial tubes;" an error respecting the anatomical characters of the disease which is repeated in the descriptions of fig. 5, plate iii.

Were we to particularize any of the drawings as more characteristic than others of the morbid appearances represented in these fasciculi, we would refer to those met with in the intestines in dysentery and in some forms of typhoid fever. Fig. 1, plate ii., third fasc., represents a remarkable case of lumbrici, contained in the biliary ducts and gall-bladder. A great many of these worms, of considerable size, are seen coiled up in the latter organ; and it is stated that "in some places they had *penetrated* through the ducts and the substance of the liver, into the cavity of the abdomen." But the opinion that any of the entozoa of the human body give rise to perforation of organs, otherwise than by exciting inflammation and ulceration, has been shown, by recent pathologists, to be altogether erroneous. In plate i., third fasc., six drawings are given of what is denominated "a species of organic disease of the liver, of an obscure kind, and, as it is believed, not yet described." From the inspection, however, as well as from the description (which is very imperfect) of the drawings, we think we can recognize appearances which we have met with several times in some forms of cirrhosis. Indeed, the so-called tuberculated state of the liver, now known under the name of cirrhosis, is pointed out, in the description of the drawings, as accompanying the morbid condition in question, except in one of the cases; a morbid condition which is described as consisting in the presence "of numerous well-defined excavations." Having met with similar appearances only in cirrhosis, and in that state of the disease in which the portal circulation has been arrested for some time before death, we believe those shown in the drawings to be of the same nature. The blood, fibrine, and frequently an admixture of bile, is found, under such circumstances, accumulated in the portal veins; which, being removed by pressure and washing, the empty orifices of these vessels appear on the cut surface of the liver like so many cells; thus giving rise to appearances similar to those represented in the drawings. Plates ix. and x. contain a number of interesting illustrations of the appearances which accompany the reparation of bone after fracture, and more especially the peculiarities of this process in fracture of the patella, produced experimentally in animals; and also some of the leading phenomena of necrosis, the results of experiments made by Mr. Gulliver.

We feel pleasure in offering our meed of praise to the officers of the medical department of the army at Chatham, who have been engaged in the publication of these fasciculi. The researches necessary to the collecting and preparing the materials for such an undertaking require much time, and the sacrifice of more agreeable and even profitable pursuits. We hope the undertaking will meet with sufficient encouragement to enable the authors to proceed with their illustrations of the valuable collection of morbid preparations contained in the Army Medical Museum, which we are glad to find is increasing both in extent and usefulness.

